# Seismic Response Evaluation of High-Steep Slopes Supported by Anti-Slide Piles with Different Initial Damage Based on Shaking Table Test

**DOI:** 10.3390/ma15113982

**Published:** 2022-06-03

**Authors:** Hongyu Chen, Guanlu Jiang, Xinhui Zhao, Dan Zhu, Yong Liu, Hongcheng Tian

**Affiliations:** 1 School of Civil Engineering, Southwest Jiaotong University, Chengdu 610031, China; 2015200219@my.swjtu.edu.cn (H.C.); 18257176909@163.com (X.Z.); zhudandanwork@163.com (D.Z.); 2 Key Laboratory of High-Speed Railway Engineering, Southwest Jiaotong University, Ministry of Education, Chengdu 610031, China; 3 China Railway Eryuan Engineering Group Co., Ltd., Chengdu 610031, China; liuyong31@ey.crec.cn; 4 POWERCHINA Chengdu Engineering Corporation Limited, Chengdu 611130, China; 2020053@chidi.com.cn

**Keywords:** earthquake dynamic response characteristics, anti-slide pile, initial damage, shaking table test, frequency change rate

## Abstract

In order to study the instability development process of the slope reinforced by anti-slide piles under earthquake conditions, the dynamic response characteristics of the slope are usually taken as the main characteristics, and the model test and numerical simulation are the main research methods. In this paper, a shaking table model test is designed and completed to investigate the influence of anti-slide piles with different initial damage on the failure mode of high and steep slope under earthquake conditions. The changes in velocity, strain and natural frequency during slope vibration are tested in combination with cloud maps when sinusoidal waves of different accelerations with a peak value of 5 Hz are applied. Thus, the differences of slope failure development process and dynamic response characteristics are obtained. The experimental results show that the anti-slide pile with different initial damage has obvious influence on the slope instability process. Under the condition of good anti-slide pile quality, the failure development of the slope behind the pile is limited to soil sliding on top of the slope, slope sliding and overburden sliding; the front slope foot of pile mainly forms shear belt and local sliding. With the decrease in the initial mass of the anti-slide pile, the slope failure develops into topsoil sliding, slope sliding and deep integral sliding; analogously, the failure of the slope in front of the pile develops into a whole slip along the slip belt. The natural frequency cloud map can directly reflect the damage location of the slope, and the frequency change rate is positively correlated with the cumulative shear strain. It shows that the macro-failure characteristics of the model slope change well when the natural frequency is used as the sensitive index to measure the influence of vibration on the model slope. The threshold value of the natural frequency change rate can distinguish different development stages of the slope; 1% is the threshold value of stage II, and 1.5% is the threshold value of stage III.

## 1. Introduction

As a kind of slope retaining structure, the anti-slide pile is widely used in railways and expressways because of its strong anti-slide ability, small disturbance to the sliding body, flexible pile arrangement and easy construction. In order to study the instability development process of a slope reinforced by anti-slide piles under earthquake action, the dynamic response characteristics of the slope are usually taken as the main characteristic quantity, and the model test and numerical simulation are the main research methods.

Previous studies [1,2,3,4,5,6,7,8] explore the interaction between piles and soil, and vibration table or centrifugal shaking table tests are used to study the dynamic response of various slopes reinforced by anti-slide piles. Although the centrifuge test has the advantage of simulating the gravity field of the prototype structure, the size effect of soil particles and anti-slide piles has not been well simulated. However, although the large-scale shaking table test cannot satisfy the similarity of the gravity field, it can analyze the interaction between structure and soil by satisfying the similarity ratio of some main variables.

Recent studies focus on the dynamic responses of different types of slopes reinforced by various anti-slide piles: Lai et al. [9] carried out the shaking table test and numerical analysis of the slope, and the study showed that the acceleration response of the double-row pile retaining gravel soil slope was more sensitive to the generation of slope cracks. Based on a large-scale shaking table test and numerical calculation, Zhang et al. [10] proved that the dynamic acceleration amplification effect of the anti-slide pile reinforcement of a soft rock high slope under different earthquake effects was related to the amplitude of ground motion. Sun et al. [11] described several characteristic quantities, such as the peak acceleration (PGA) amplification coefficient, acceleration response spectrum and vertical settlement deformation in order to study the seismic response characteristics of the underlying bedrock accumulation slope before and after the reinforcement of anti-sliding row piles. Zhang et al. [12] studied the seismic response characteristics of the slope of the bridge foundation reinforced by anti-slide piles through a shaking table test, and proposed that the peak value of the acceleration response was correlated with the peak value of the specific frequency band of the input seismic wave based on the acceleration response spectrum. Gischig et al. [13] obtained that the amplification coefficient of slope frequency is highly sensitive to the change of cracks by using the numerical simulation method. For other retaining structures, such as the pile-anchor structure, micro-pile and so on, the time-frequency domain analysis based on slope acceleration has also become a means of analyzing slope dynamic response [14,15,16,17,18,19,20,21,22,23,24,25,26,27,28,29].

The dynamic response of the slope strengthened by anti-slide piles is mainly to analyze the acceleration amplification effect and the change of the related frequency spectrum, and to analyze the development process of slope instability combined with the test phenomenon. However, the instability of slope is actually due to the development of the local strain of soil, and the instability of slope under dynamic action is mainly due to the development of shear strain in the slope, which results in plastic flow. There are few studies on shear strain measurement inside a slope. Previous studies [30,31,32] used distributed optical fiber to monitor slope strain. For laboratory tests, there are few researches on soil-related strain monitoring methods that are easy to operate and convenient. Sato et al. [33] proposed the method of using phosphor bronze belt to measure the shear deformation of surrounding soil, which was successfully used by Fu et al. [34] and Lei et al. [35] to measure soil shear strain in shaking table test, but the development law of soil shear strain has not been comprehensively analyzed in combination with other dynamic response indexes.

The anti-sliding piles involved in the above investigation are all the dynamic response characteristics of the pile or slope when the pile body is not damaged. When there is damage to the pile body, the dynamic response law under the same load must be different compared to when there is no damage. The accumulation of pile structural damage is involved in the process of anti-slide pile bearing load [36]. In the initial pouring and subsequent loading process of the anti-slide pile, damage accumulation of the pile body structure may occur in different degrees. The influence of the damaged anti-slide pile on slope deterioration is the key to evaluate slope stability under multiple loads. There is a lack of research on the influence of an anti-slide pile with different initial damage degrees on slope instability development under dynamic action. Shaking table tests of anti-slide piles supporting high and steep slopes with different initial damages are designed and completed. The differences of acceleration amplification response, shear strain development and natural vibration frequency with different initial damage of anti-slide pile are analyzed, and the rationality of the shear strain analysis is clarified. The relationship between the change rate of natural vibration frequency and the change of shear strain is analyzed, which provides a technical reference for monitoring the deterioration of slope in the actual field. Additionally, the threshold values of shear strain and natural frequency are proposed based on experiments.

## 2. Shaking Table Test Model

The test was carried out on a unidirectional electro-hydraulic servo-driven shaking table (High-speed Railway Engineering Laboratory, Southwest Jiaotong University) with rigid model box with internal dimensions of 3.7 m × 1.5 m × 2.1 m (length × width × height). In order to reduce the reflection effect of the input seismic wave on the back wall of the model box, polyurethane foam plate was set on the back of the model box for shock absorption.

### 2.1. Similarity Theory Design

Separation design was adopted to study the influence of different pile damage degrees on slope damage development according to the test section of Lei et al. [35]. Considering the size of the model box and taking geometric size, weight and acceleration as the main controlling factors, the physical similarity constants of soil, pile and seismic wave are deduced according to the similarity criterion (see Table 1).

The actual stress–strain distribution of the soil is difficult to simulate because it cannot satisfy the similarity of gravity field. For the materials of geotechnical simulation, it is necessary to ensure that the similar relationship between stress and strain of soil is maintained in the process of vibration when the inertial force of soil is investigated. The damping ratio of soil meets the similarity ratio when the dynamic shear modulus ratio of the material meets the similarity ratio. In this way, the nonlinear characteristics, stress–strain hysteresis and plastic deformation accumulation of soil in terms of dynamic response are guaranteed to meet the similarity relationship [35]. The reference strain value in Stokoe model was used as the similarity evaluation index of dynamic shear modulus ratio changing with shear strain, which was verified [37,38]. Therefore, the semi-quantitative analysis of slope strain development law in this paper is only applicable to the slope whose reference strain value is similar to that of the soil in this paper.

### 2.2. Design of Damaged Pile

The essence of anti-slide pile damage is the reduction of pile stiffness, which can be divided into local stiffness damage and overall deterioration damage. The macroscopic phenomenon of local stiffness damage is that cracks occur in the pile body during production or loading. It can be simulated by setting cracks at the joint with the maximum stress of the pile. The overall deterioration damage can be achieved by reducing the cross-section size of the pile or using low-strength pile material; this was simulated by setting up pile cavity in this test.

The maximum position of shear force and bending moment along the pile body is the key node for the deterioration of the performance of the anti-slide pile. Once the crack continues to develop, the retaining capacity of the anti-slide pile will further weaken. In the study of different types of high-steep slopes, it is found that the soil pressure on pile body presents a triangular distribution under static action, and the maximum value is located in the middle of slide body. The bending moment is s-shaped, and the maximum value is located near the rock–soil interface, mainly causing bending failure [39,40]. The distribution of the form of soil pressure under dynamic action may be parabolic, triangular and approximate trapezoidal. The maximum bending moment is usually located near the weak interface, which is prone to bending failure [4,41].

Therefore, according to different initial damage degrees, anti-slide piles are divided into three groups: A, B and C. Initial damage arrangement is as follows:

Group A: No initial damage and no preset artificial crack in pile body;

Group B: Pile body with local initial damage that symmetrical layout artificial cracks with depth of 5 mm + 15 mm;

Group C: With local initial damage and overall deterioration damage. According to the test results of Group B, the most unfavorable position is located on the preset sliding surface, so the 5 mm + 5 mm crack position is adjusted to the rock–soil interface. Artificial cracks of 5 mm + 15 mm are still arranged in pile body, and hollow holes of 8 mm diameter are preset in order to reduce pile body quality.

**Figure 1:**
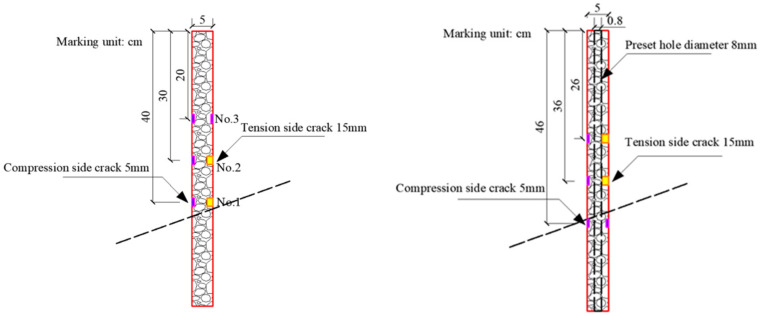
B (**left**) quality grade and C (**right**) quality grade pile (unit: cm).

Piles of grade B and C are shown in Figure 1. The bending stiffness EI of pile is calculated for the stiffness reduction at the crack, and the results are shown in Table 2.

### 2.3. Model Filling and Instrument Layout

**Figure 2:**
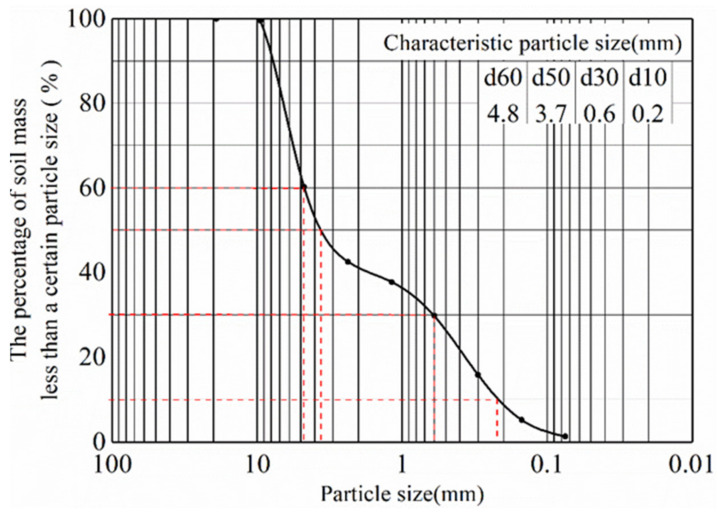
Sliding mass gradation.

**Figure 3:**
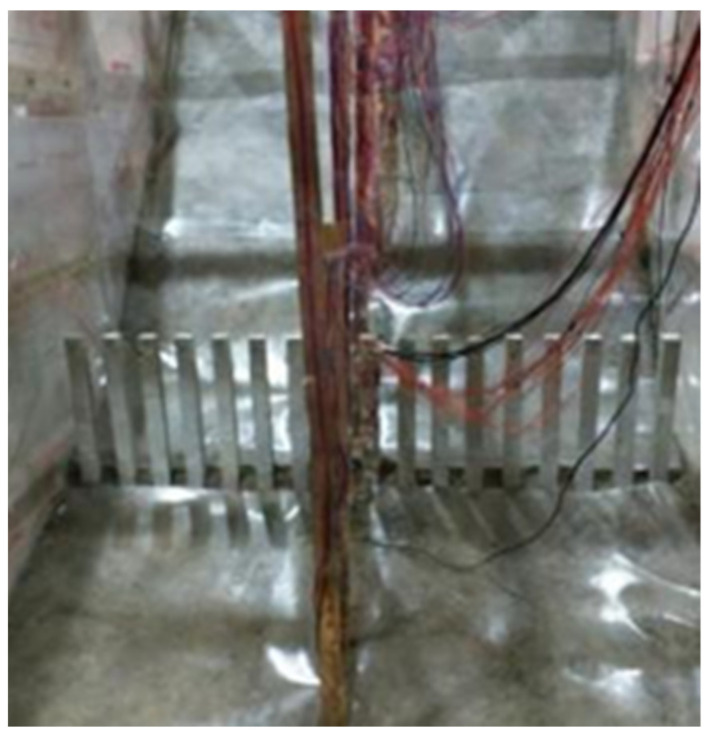
Slide-belt landfill.

**Figure 4:**
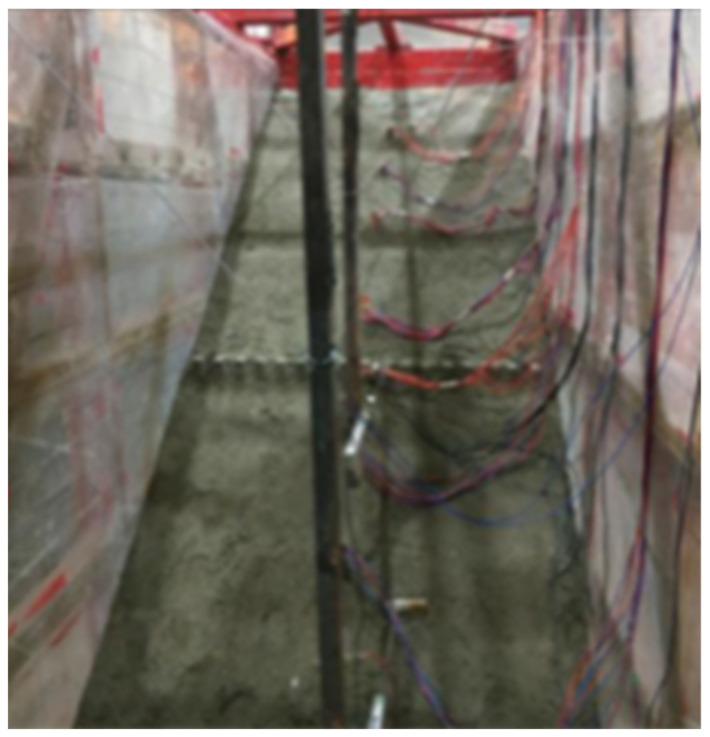
Slope landfill.

Refer to previous studies of Fu et al. [34] and Lei et al. [35]. The concrete of anti-slide pile is made according to the standard ratio of C35 grade, and the detailed design parameters are shown in Table 3. Under the premise of meeting the strength requirements, the red clay widely distributed in Chengdu area was selected as the bedrock and mixed with a certain proportion of gravel, fine sand, cement and early strengthening agent. In order to ensure that the sliding body can produce the maximum sliding force under earthquake action. An artificial sliding surface, which is a plastic film with a thickness of 2 mm, is set up between the sliding body and the bedrock, and a little silicone oil is applied for lubrication. The sliding body was simulated using a mixture of sand and gravel, and the grading of gravel soil layer is shown in Figure 2. Physical and mechanical parameters of model materials are shown in Table 4. Slip belt filling and slope model filling are shown in Figure 3 and Figure 4.

The size of the test model is 3.5 × 1.5 × 2 m^3^. The anti-sliding section has a gentle slope of 19°, and the sliding section has a steep slope of 46°. The slope of the sliding body is about 36°. The section size of anti-slide pile is rectangle of 5 × 3.6 cm^2^. Pile length of 66 cm, laid 110 cm away from the foot of the slope. The pile spacing is 8.6 cm, and a total of 17 piles are laid in cross section.

**Figure 5:**
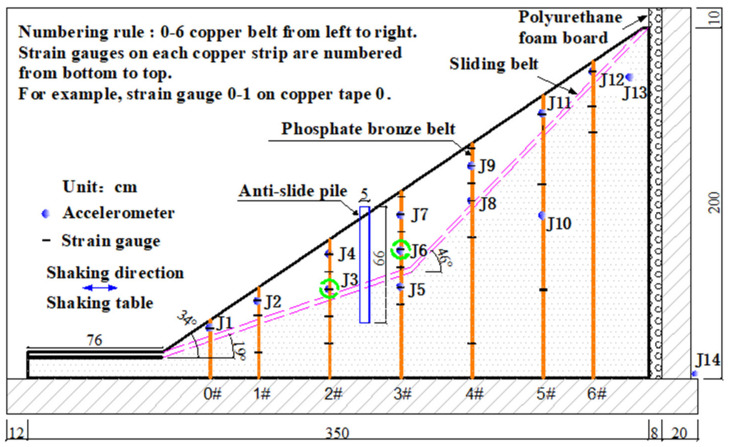
Test components layout.

Various instruments are embedded inside the slope to monitor the dynamic response of the slope body. The specific instrument layout scheme is shown in Figure 5. Accelerometer is used to monitor the change of acceleration of the soil; phosphor bronze strip with strain gauge is used to measure the change of shear strain of the soil.

### 2.4. Loading Scheme

**Figure 6:**
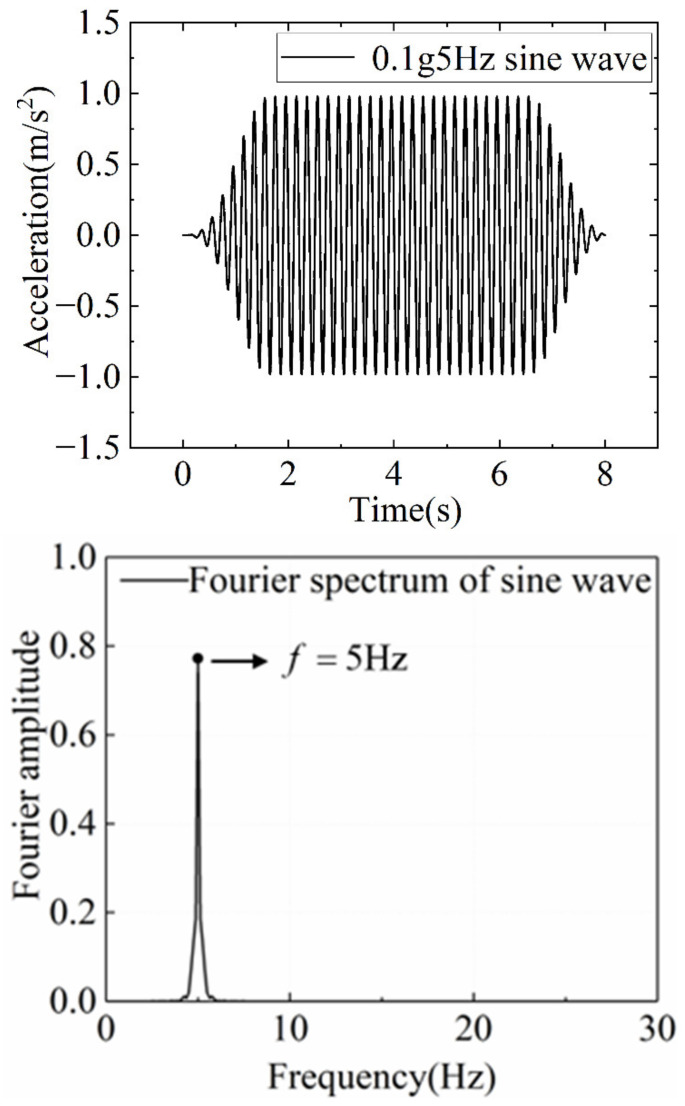
5 Hz sine wave waveform and Fourier spectrum.

**Figure 7:**
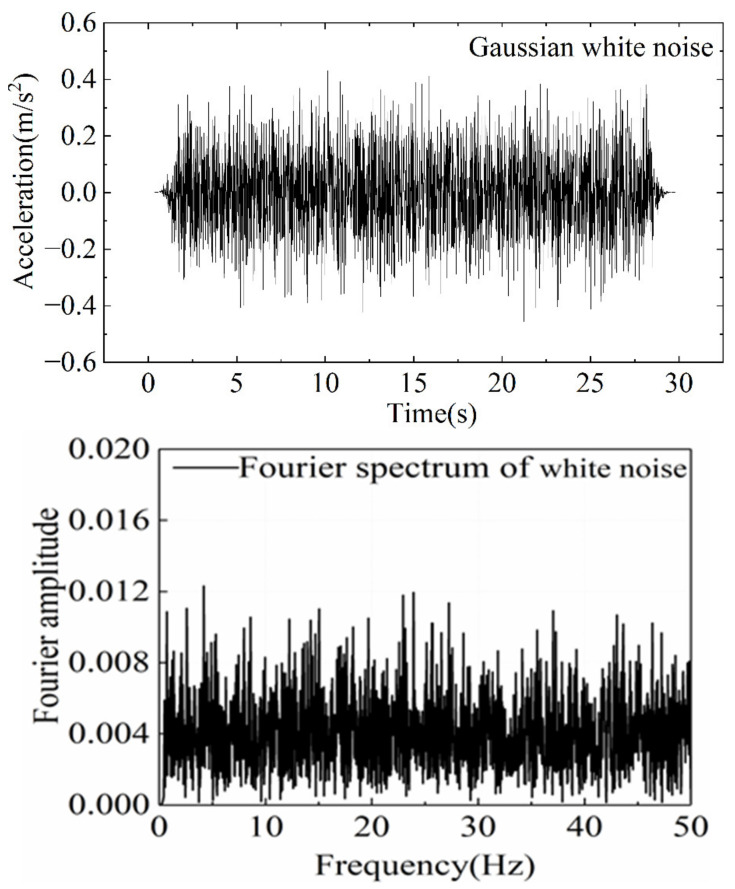
White Gaussian noise waveform and Fourier spectrum.

In order to analyze the dynamic response of the slope reinforced by anti-slide piles with different levels of initial damage, the 5 Hz sine wave is used as the applied dynamic load, and its peak acceleration increases gradually from 0.1 to 0.6 g. Then, the 0.6 g sine wave was repeatedly loaded until the model was damaged. In addition, 30 s of Gaussian white noise sweep was applied before and after each sine wave loading to obtain the natural frequency characteristics of slope. The loading sequence is shown in Table 5, and the shape and Fourier spectrum of loading wave is shown in Figure 6 and Figure 7.

## 3. Results and Analysis

### 3.1. Test Phenomenon

**Figure 8:**
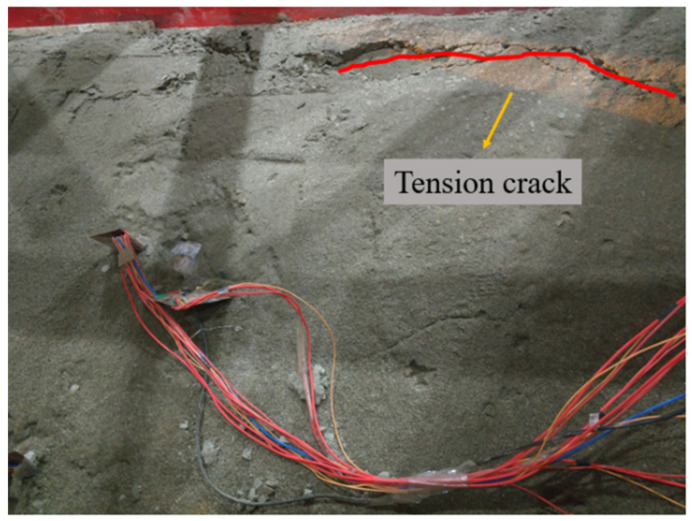
Slope top after 0.6 g sine wave first loading.

**Figure 9:**
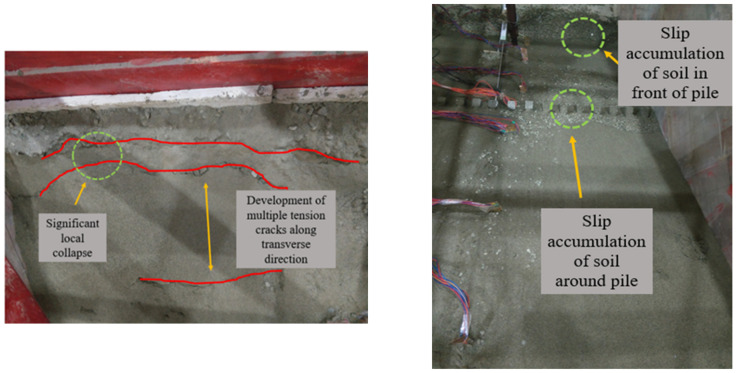
The top, middle and toe of the slope after 0.6 g sine wave 4th loading.

For group A, only a small amount of sand and gravel in the model slipped along the slope during the loading process of the 0.1~0.5 g sine wave. As shown in Figure 8 and Figure 9, after the first loading of the 0.6 g sine wave, tensile cracks began to appear at the top of the slope due to the increasing tension at the back edge of the slope. After the fourth loading of the 0.6 g sine wave, many tensile cracks on the top of the slope continued to develop laterally, leading to obvious layered collapse of local soil. However, only surface slip occurred in the slope body at this time, and the overall stability of the slope was still good.

**Figure 10:**
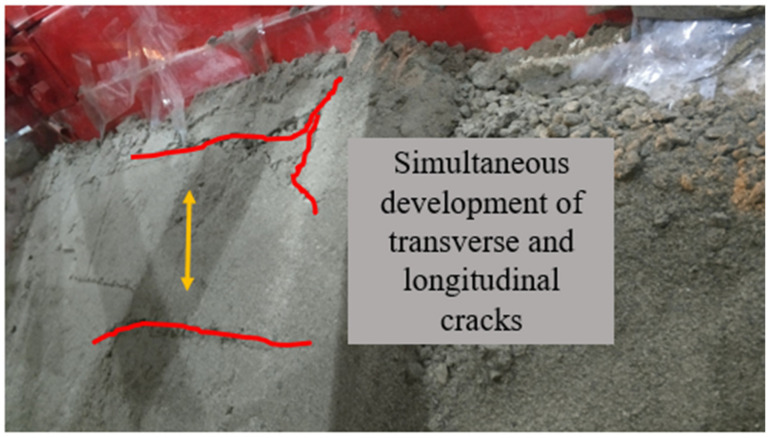
Slope top after 0.5 g sine wave loading.

**Figure 11:**
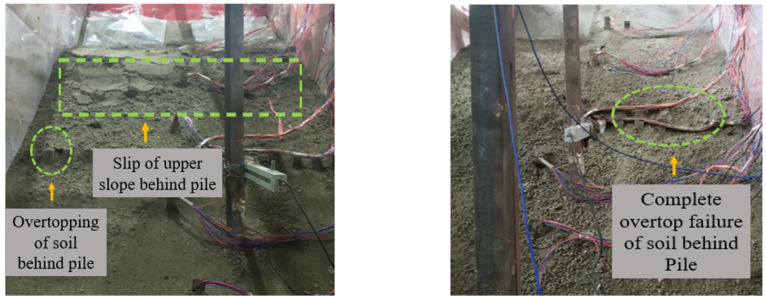
The 2nd (**left**) and 4th (**right**) loads of 0.6 g sine wave.

For group B, the phenomenon of 0.1~0.4 g sine wave loading was similar to that of group A, and the slope was relatively stable. After the sine wave reached 0.5 g, several longitudinal and transverse tensile cracks appeared at the top of slope (Figure 10 and Figure 11). After the fourth loading of the 0.6 g sine wave, compared with the surface collapse of group A, group B had witnessed the failure of the slope body behind the pile over the top, and the whole slope body slipped and collapsed.

**Figure 12:**
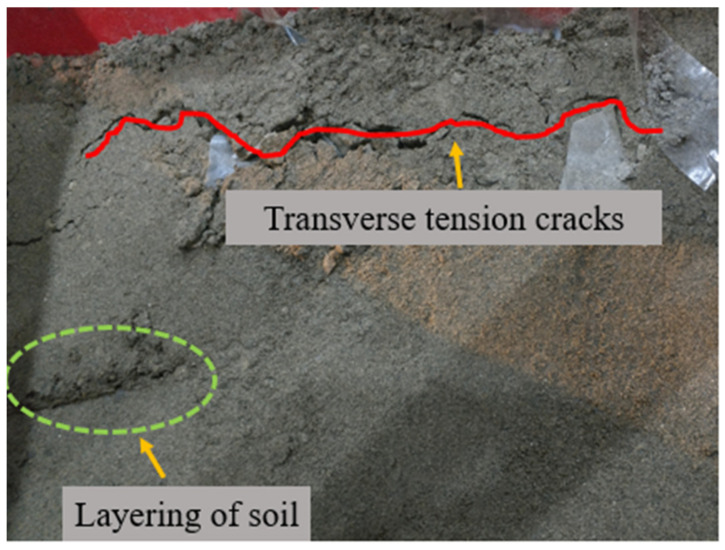
Slope top after 0.4 g sine wave loading.

**Figure 13:**
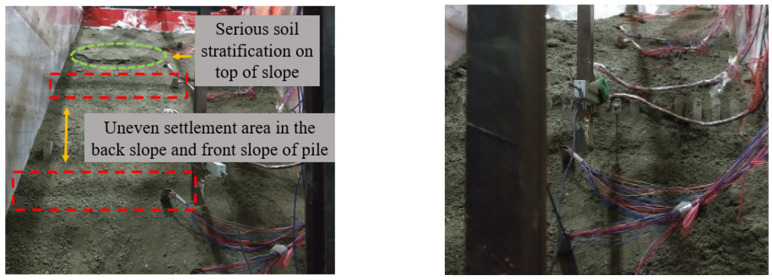
0.6 g sine wave 1st (**left**) and 3rd (**right**) loads.

The test phenomena of group C are shown in Figure 12 and Figure 13. The phenomenon of 0.1~0.3 g sine wave loading is similar to that of group B, and the slope is relatively stable. Tensile cracks appeared at the top of the slope after the loading of the 0.4 g sine wave. After the first loading of the 0.6 g sine wave, an obvious uneven settlement area was generated in group C in both the back slope of the pile and the front slope of pile compared with group B. The reason for this is that there are obvious differences in the motion modes of deep soil before and after the region under the action of this magnitude, that is, displacement discontinuities are produced. It is suggested that in addition to surface slip, there was also overall slip in the depth of soil relative to the slope, resulting in uneven settlement. After the third loading of the 0.6 g sine wave, the soil failed over the top and the slope collapsed completely.

Compared with the three test phenomena, the failure mode of the slope is that the tensile cracks gradually occur at the top of slope. With the increase in earthquake magnitude, the slope sliding failure gradually developed to the lower part. The upper soil produced slope sliding collapse and over-top failure. The smaller the damage of anti-slide pile, the more surface development of slope failure. With the increase in initial pile damage, the slope damage gradually moves down along the depth while the slope surface slips and collapses. The test phenomenon shows that the settlement and slip of the slope body are not uniform and obvious stratification occurs.

### 3.2. Slope Acceleration Analysis

According to the measured PGA amplification coefficient and corresponding coordinates at the acceleration measurement points J1~J13 inside the model, the two-dimensional PGA amplification coefficient matrix of the model was obtained by interpolation fitting. The PGA amplification coefficient nephogram of the model was drawn, and the law of the PGA amplification coefficient of the model with different peak sine loadings is obtained. Thus, the dynamic response state of the model is summarized and judged.

**Figure 14:**
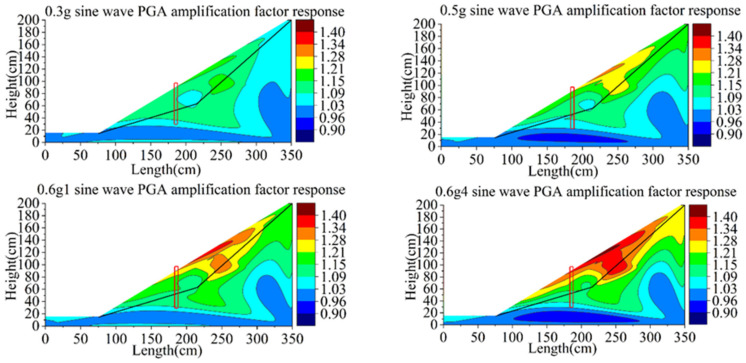
Contours of PGA amplification coefficient of model A.

The nephogram of the PGA amplification coefficient of group A is shown in Figure 14. The overall vibration response of the model becomes more and more intense with the increase in loading peak value. The PGA amplification coefficient of the sliding body is larger and at the range of 1~1.4, while that of bedrock is smaller and at the range of 1~1.1. The amplification coefficient of PGA at the inner end of the anti-slide pile and 1/2 of the slope is the largest, indicating that the slope vibration near this region is the strongest in the whole model and the inertia force is larger. The PGA amplification coefficient is small at the slope foot, the turning point of the sliding surface and the slope top near the back wall of the model box. This may be because the horizontal angle variation of the slope foot and sliding surface and the boundary of model have a certain attenuation effect on the horizontal (x direction) wave input.

According to the test phenomenon, at the first loading of 0.6 g sine wave, tension cracks appear at the top of the slope, but the PGA amplification coefficient does not reflect this change.

**Figure 15:**
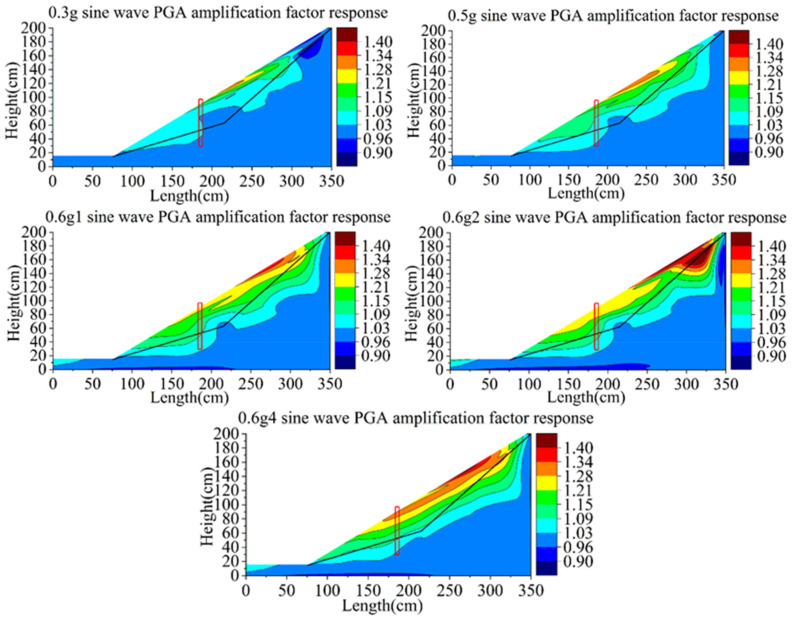
Contours of PGA amplification coefficient of model B.

The PGA amplification coefficient nephogram of group B is shown in Figure 15, the distribution of which is basically consistent with group A. Vibration is weakened at the foot of the slope, the turning point of the sliding surface and at the top of the slope near the back wall of the model box. The PGA amplification coefficient of bedrock range is 1~1.1 and increases slightly with the increase in height. When loading the 0.5 g sine wave, tension cracks began to appear at the top of the slope, but PGA has no mutation, so the PGA response is not sensitive to model damage. When the second loading of the 0.6 g sine wave occurs, the upper part of the sliding body has slipped, the accelerometer at the top of the slope is exposed, and the PGA response increases. The 1/2 part of the slope from the top of the pile to the top of the slope is still the most intense vibration area. After the fourth loading of the 0.6 g sine wave, the slope failure completely occurred, and the PGA amplification coefficient of the whole slope surface basically kept increasing along with the height of the slope, but it attenuated near the boundary of the model.

**Figure 16:**
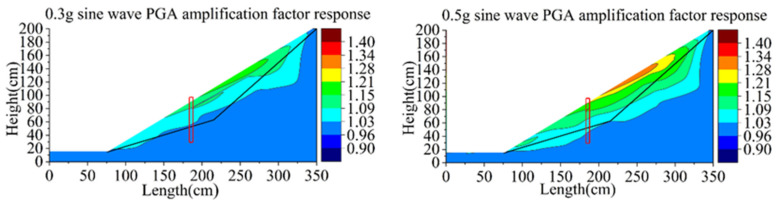

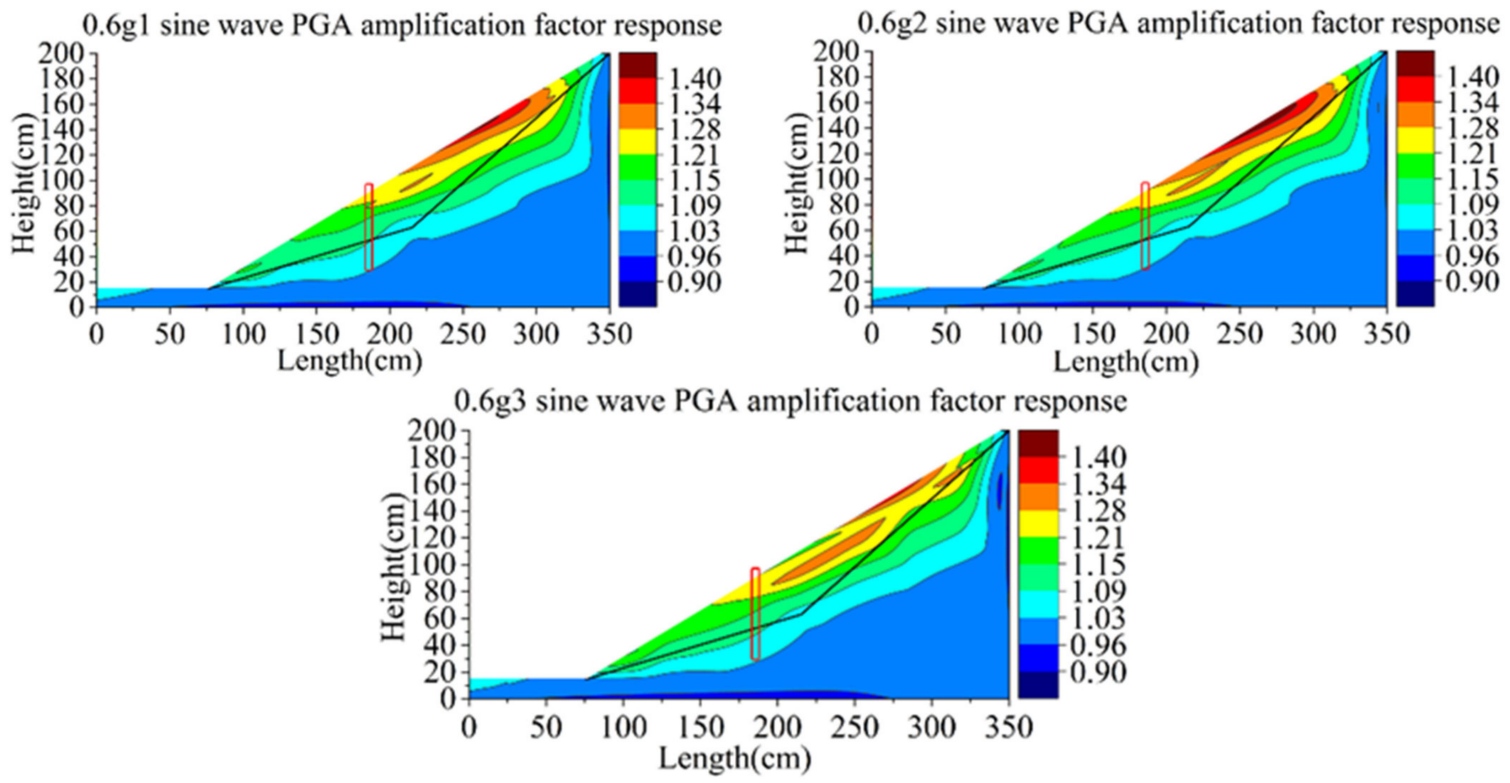
Contours of PGA amplification coefficient of model C.

The PGA amplification coefficient nephogram of group C is shown in Figure 16. Compared with the first two groups, the initial damage of the anti-slide pile in group C is the largest, the pile strength is the lowest, and the slope failure occurs earlier.

By comparing the PGA nephogram of the three groups of tests, the 1/2 position of the slope behind the pile is the most intense vibration area. Acceleration can only represent the motion of soil, and the slope is less constrained due to the free-face amplification effect. Therefore, acceleration is sensitive to the development of failure of the soil on the slope, but it is not sensitive to the development of failure of the soil under the slope.

**Figure 17:**
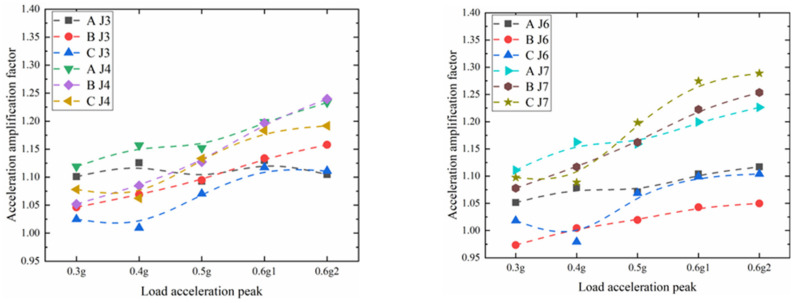

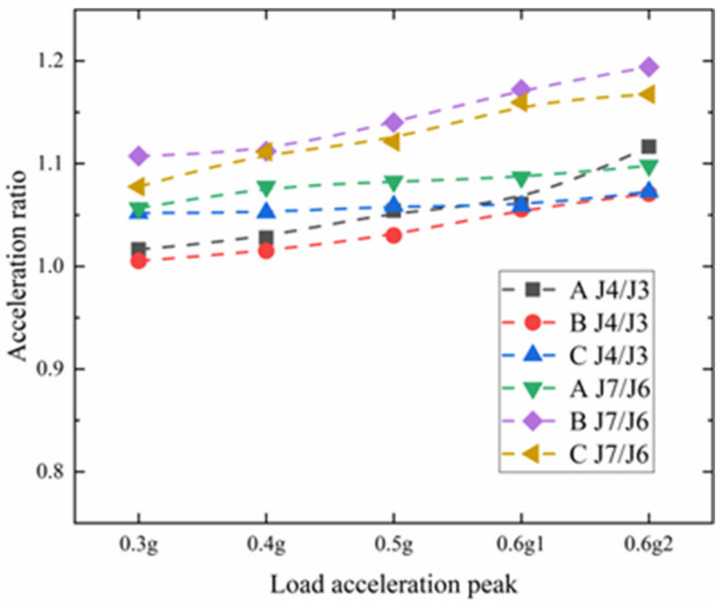
Comparison of three groups of acceleration amplification factors.

Compare the acceleration amplification factors of the corresponding J3, J4, J6, and J7 measuring points before and after the pile (Figure 17). For the position in front of the pile, J3, near the sliding surface, when the pile body is complete (group A), the acceleration amplification factor changes little, all around 1.10, indicating that the soil damping change near the sliding surface in front of the pile is small. When the initial damage is small (group B), the initial acceleration amplification factor of the pile body is 1.05. As the magnitude increases, the acceleration amplification factor increases linearly, the pile generates displacement, the local soil in front of the pile becomes dense, and the damping decreases. At 0.6 g, it increased to 1.15, and when there was a large amount of initial damage to the pile body (group C), the acceleration amplification factor showed an obvious law of first slightly decreasing, then increasing, and then stable. Finally, the acceleration amplification factor only reached 1.1. For J4 near the surface, due to the influence of the elevation amplification effect, the law is similar to that of J3, the value is larger than that of J3, and the slope becomes larger.

Compared with point J3 before the pile, the acceleration amplification factor of J6 be- hind the pile is between 1.05 and 1.10, which is smaller than that of J3 before the pile, indicating that the anti-sliding pile has a greater effect on the sliding limit of the soil near the sliding surface behind the pile than the soil before the pile, and the local damping reduction speed is slower than that near the front sliding surface of the pile. For J7 behind the pile, the two groups of A and B are similar to J4 before the pile, and the maximum values are between 1.23 and 1.25. Compared with groups A and B, J6 of group C was significantly increased, while that of J4 on the front slope of the pile was significantly decreased. It shows that when the initial quality of the anti-sliding pile is poor, the soil on the slope behind the pile will undergo local slip accumulation, resulting in a decrease in the damping of the soil near the slope behind the pile, and an increase in the PGA nonlinearity. However, the position of J4 in front of the pile is located on the free surface, and the anti-sliding pile cannot provide sufficient energy dissipation effect. Under the action of the most unfavorable acceleration, the soil on the slope surface repeatedly shears and dissipates energy, resulting in the obviously decreasing acceleration amplification factor trend relative to the A and B groups. 

Comparing the two sets of acceleration measuring points, J3, J4 and J6, J7, with a height difference of 20 cm, before and after the pile, divide the measured acceleration and study the difference of the magnification effect along the elevation at different positions. Under the same damage degree, the elevation amplification effect of the front and rear of the pile increases gradually with the increase in the magnitude. With the increase in the initial damage of the pile body, the slope of the acceleration ratio in front of the pile gradually becomes smaller, indicating that the larger the initial damage is, the height amplification effect of the front of the pile is not greatly affected by the magnitude; the slope of the acceleration ratio behind the pile increases with the initial damage of the pile body This gradual increase indicates that the initial damage is greater, and the height amplification effect of the pile trailing edge is continuously increasing due to the influence of the magnitude. With the increase in the initial damage of the pile body, the slope of the acceleration ratio in front of the pile gradually becomes smaller, indicating that the larger the initial damage is, the height amplification effect of the front of the pile is not greatly affected by the magnitude. The slope of the acceleration ratio behind the pile gradually increases with the increase in the initial damage of the pile body, indicating that the larger the initial damage, the more the height amplification effect of the rear edge of the pile is continuously increased by the magnitude of the impact.

### 3.3. Shear Strain Analysis

The actual shear deformation of soil is difficult to measure. The residual accumulative value of bending strain measured by the phosphor bronze belt under dynamic action reflects the shear deformation of soil indirectly. Thus, the sliding position at the beginning and the development trend of the deformation of slope under dynamic action are revealed.

**Figure 18:**
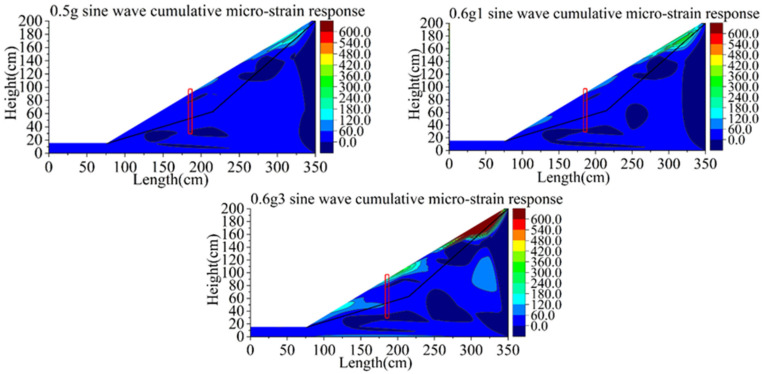
Contours of shear strain response of model A (unit: με).

Figure 18 shows the nephogram of the shear strain response of Group A. The integrity of the model is good in the whole loading process. At the load of the 0.5 g sine wave, the slope begins to slide. When the first loading of the 0.6 g sine wave occurs, tensile cracks appeared at the top of the slope, local slip began to occur, and shear bands appeared at the front slope foot of the pile. After the third loading of the 0.6 g sine wave, a small local collapse occurred at the top of the slope, and a sliding trend occurred at the local slope surface behind the pile, but the overall stability of the slope was still good. The fastest developing area of shear strain is mainly concentrated at the top of slope, and the magnitude of strain of bedrock is very small. It shows that the main failure mode of the rear slope is a partial collapse at the top of the slope when the pile quality is intact, which mainly concentrates on the slope surface, and no deep sliding occurs. The failure mode of the pile front slope is mainly caused by the formation of a local shear belt at the slope toe.

**Figure 19:**
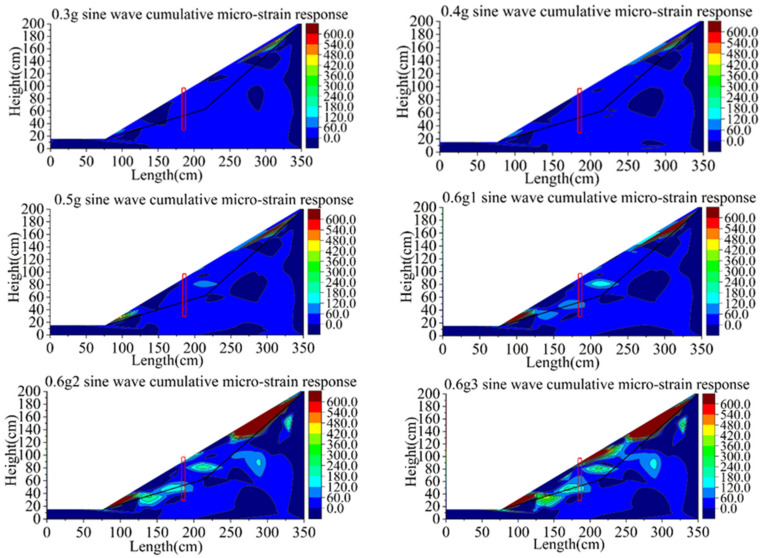
Contours of shear strain response of model B (unit: με).

Figure 19 shows the cloud diagram of the shear strain response of group B. The strain development of the soil of group B is similar to that of group A at the 0.3~0.4 g sine wave. With the increase in the loading peak value, the shear strain near the top of the slope increases. When the sine wave reached 0.5 g, tension cracks appeared at the top of the slope, the shear strain increased sharply and shear bands gradually formed near the toe of the slope. Large shear strain occurs locally at the near-end of the sliding surface behind the pile. The reason is that this position is just at the folding angle of the sliding surface and close to the pile, acting as a retaining structure on the soil of the far-end behind the slope together with the pile. Therefore, the strain at the near-end behind the pile is also developing, but the strain near the sliding surface in the middle of the slope is small. At the first loading of 0.1 g, the shear strain at the top of the slope and behind the pile develops further, and the shear strain in front of the pile develops rapidly along the slip belt. The soil in front of the pile developed from partial sliding at the foot of the slope to overall sliding along the sliding surface. At the second loading of 0.6 g, the soil at the top of the slope behind the pile slips along the sliding surface in a large area and approaches to failure over the top. The shear strain was along the sliding surface on the front of the pile run through, indicating that the soil in front of the pile had started to slip as a whole, and the soil at the slope foot had also started to scatter. After the third loading of 0.6 g, the slope collapsed completely, and the anti-slide pile support structure failed. The over-top failure of the slope body behind the pile mainly occurred along the slope surface and did not form a whole slip along the sliding surface.

Because the anti-slide pile of group B with slight initial damage, the slope support effect of group B is obviously less than that of group A. The main failure modes of soil behind pile are slipping of slope top and over-top failure of the near-end of the pile. Compared with group A, the failure range of group B gradually develops towards the sliding surface, but there is no overall sliding along the sliding surface. The slope toe of soil in front of the pile partially slips, and the shear strain develops gradually along the sliding surface until it runs through to form the sliding crack surface, and the whole instability occurs. There was no overall slip along the sliding surface in group A.

**Figure 20:**
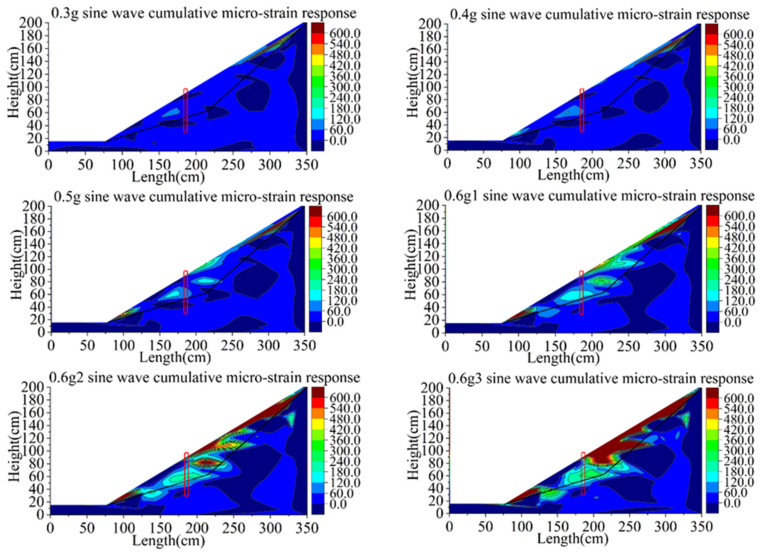
Contours of shear strain response of model C (unit: με).

The strain change of group C is more obvious than that of the first two groups (Figure 20). At the loading of the 0.4 g sine wave, the strain near the sliding surface on the slope top begins to change greatly, and the corresponding phenomenon is the formation of tension crack. At the sine wave of 0.5 g, large shear strains occur on the slope top, the slope surface at the near-end behind the pile and the slope toe, which is similar to group B. At the first loading of 0.6 g, the shear strain belt has been formed on the slope behind the pile, and the depth of shear strain development is closer to the sliding surface than that of group B. The trend of failure development of the slope in front of piles is the partial sliding of slope toe and deep sliding along the sliding surface, similar to group B. The development trend of slope failure under the second and third loading of 0.6 g is similar to that under the first loading of 0.6 g.

By comparing the development of the shear strain of high-steep slopes supported by anti-slide piles with different initial damage, the development rate of the slope failure affected by the initial damage of the anti-slide pile is determined. Although the difference in bending stiffness between group B and group C is small, the slope shear strain of group C with overall damage develops faster than that of group B with local damage.

The initial damage of the anti-slide pile also has great influence on the failure mode of the back slope. The development of the shear strain of the slope begins at the top and foot of the slope.

The anti-slide piles of different qualities have an effect on the process of slope instability. When the quality of the anti-slide pile is intact, the failure development of the slope behind the pile is limited to the soil landslide at the top of the pile. The local slip of the shear belt in front of the pile mainly occurs at the foot of the slope. With the decrease in anti-slide pile mass, the failure of soil behind the pile changes into landslide, slope sliding and deep integral sliding. The failure in front of the pile develops into local slip in the toe shear belt, and then the whole slip occurs along the slip belt. 

### 3.4. Analysis of Natural Vibration Frequency

Because the test of group A was relatively stable, no great damage occurred, and the change of natural vibration frequency was small. Therefore, group B and C with greater initial damage are analyzed. Models of group B and C are swept with Gauss white noise before and after action of the sine wave. The transfer function method [42] was adopted to determine the change of natural vibration frequency at the position of the acceleration sensor J1–J13 through the peak value of the imaginary part. The nephogram of slope frequency variation of each loading condition is shown as follows.

**Figure 21:**
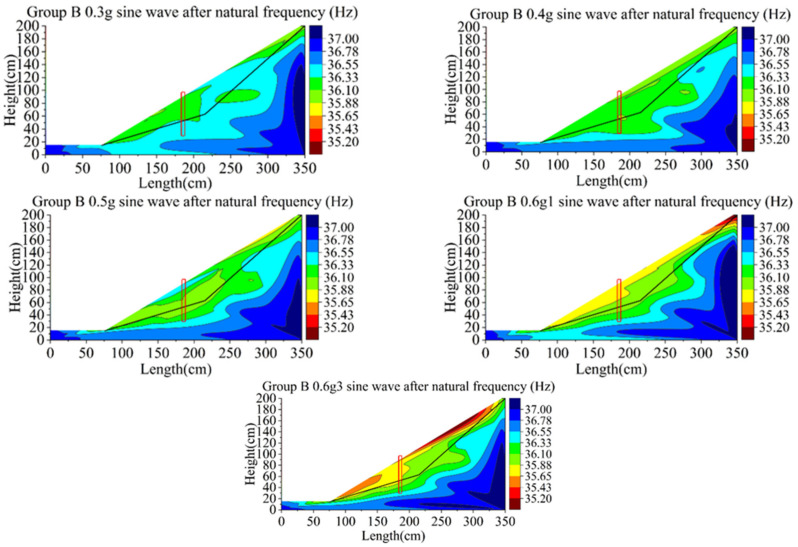
Contours of natural frequency of model B.

**Figure 22:**
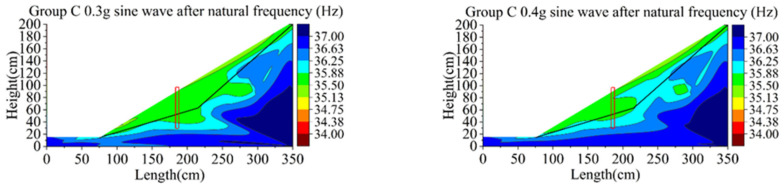

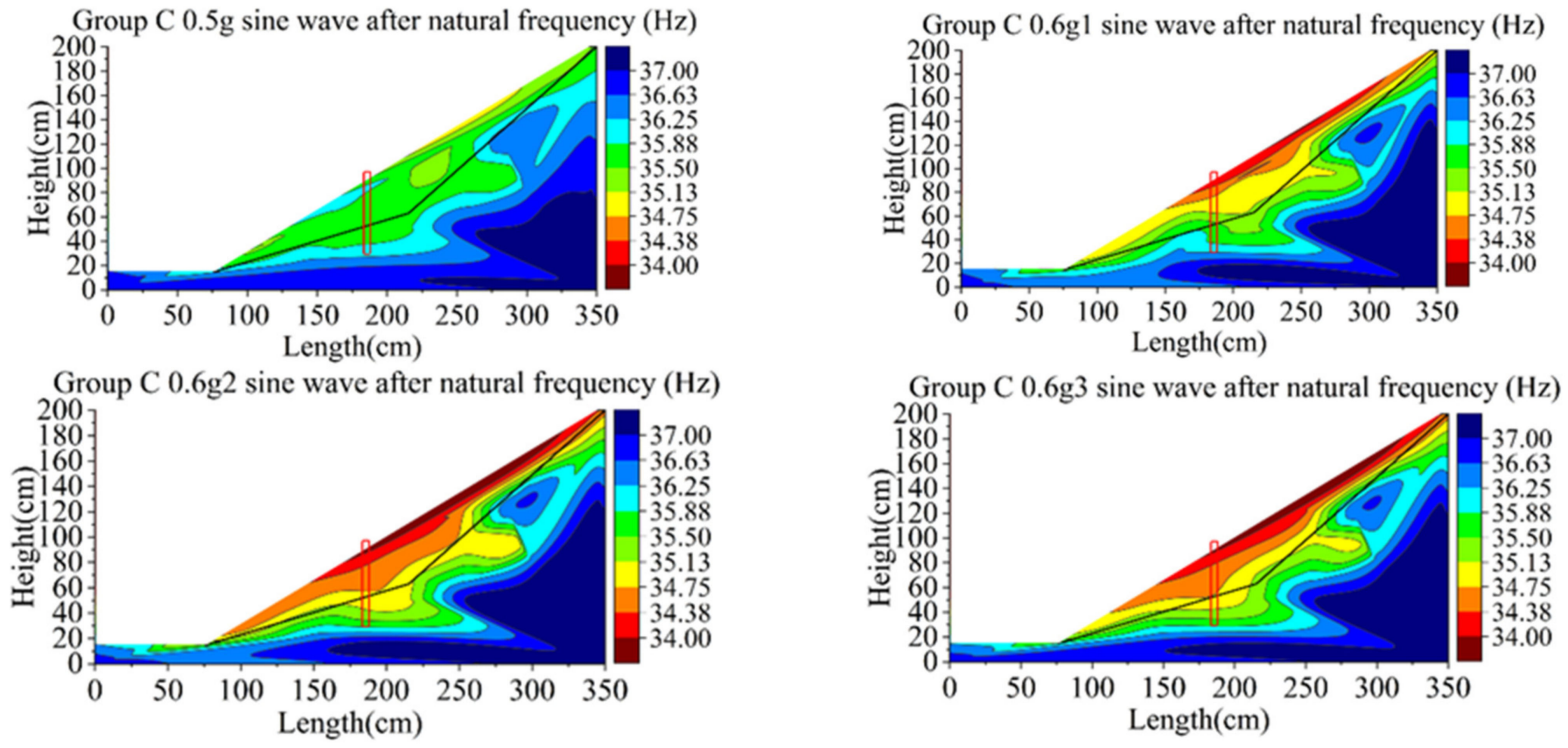
Contours of natural frequency of model C.

Figure 21 and Figure 22 show the changes of natural vibration frequency of group B and C. The initial natural vibration frequency of the sliding body in group B is mainly around 36Hz, and that of bedrock is 37 Hz. The natural vibration frequency of the sliding body decreases from the maximum 37 to 34 Hz (decrease in 8.1%) in the process from initial state to the third loading of the 0.6 g sine wave. The initial frequency of the sliding body in group C is similar to that of group B, and the bedrock is also 37 Hz. After the third loading of the 0.6 g sine wave, the natural vibration frequency of the sliding body decreased from 36 to 33 Hz, decreasing by about 8.3%.

As can be seen from the phenomena of group B and C, with the increase in loading peak value, the natural vibration frequency of the sliding body shows a downward trend, while that of the bedrock basically remains unchanged.

With the increase in earthquake magnitude, the soil behind the pile compresses the pile body, which makes the bearing capacity of the pile reach the ultimate failure value; accordingly, it produces large displacement and deformation, and the retaining effect is weakened. The soil around the pile becomes loose and the natural vibration frequency decreases continuously. The variation range of the natural vibration frequency of soil at the foot of the sliding body and the decrease range of the slope behind the pile are the largest. Additionally, the natural frequency nephogram can basically reflect the failure mode of the slope behind the pile developing from the slope slip to the deep integral slip. It shows that the variation of natural vibration frequency of the slope during loading can directly reflect the degree of damage caused by an earthquake.

### 3.5. Relationship between Natural Vibration Frequency of Slope and Shear Strain

In order to study the relationship between natural vibration frequency and cumulative shear strain of the slope, the data of J3 and J6 are selected as the research object to study the relationship between cumulative shear strain of the slope and rate of change of natural vibration frequency.

The calculation equation of the rate of change of natural vibration frequency is as follows:

(1) $$\lambda =\frac{f_{in}-f_{en}}{f_{in}}$$

where λ is the rate of change of natural vibration frequency, $f_{in}$ is the initial natural vibration frequency, and $f_{en}$ is the natural vibration frequency after each stage of loading.

**Figure 23:**
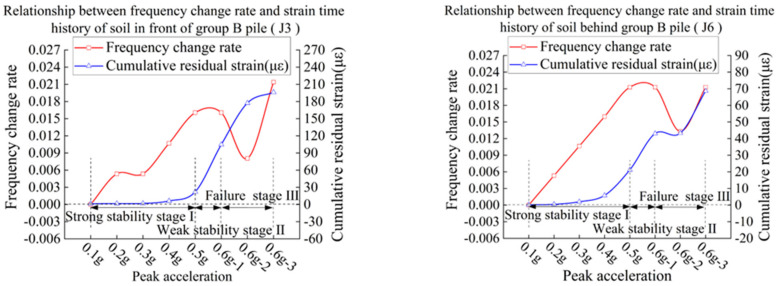
Slope cumulative strain-frequency decline rate curve of model B.

**Figure 24:**
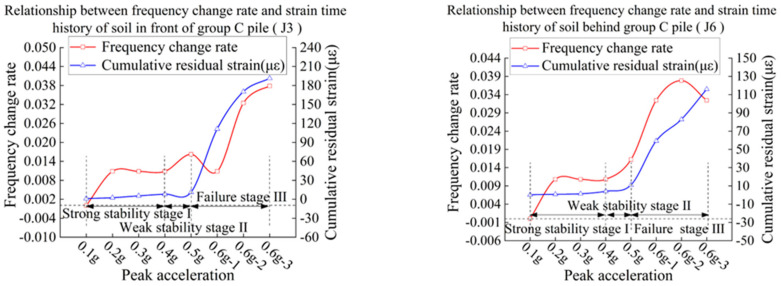
Slope cumulative strain-frequency decline rate curve of model C.

Curves of relationship of cumulative shear strain and frequency decline rate of slope in groups B and C are shown in Figure 23 and Figure 24.

Combined with test phenomena and changes of parameter, the failure behavior of slope can be divided into three stages:

Strong stability stage I: In this stage, the anti-sliding force is mainly provided by the cohesive force, which is characterized by relatively small acceleration and strain.

Weak stability stage II: At this stage, the bond force begins to decrease, and friction makes up for the insufficient slip resistance. The characteristic is that the equivalent stiffness coefficient of the bond force decreases, causing the overall natural vibration frequency of the slope body to decline correspondingly, and the cumulative shear strain continues to increase rapidly.

Failure stage III: The slope body is further disturbed and reaches the maximum static friction, but it is not enough to make up for the loss of cohesive force. The shear strain of the slope increases rapidly, which leads to failure of the slope.

According to the history curve of cumulative shear strain-frequency change rate of soil at J3 and J6, with the increase in peak acceleration and frequency, the natural vibration frequency of the model continues to decline, and the rate of each stage is similar to that of the cumulative shear strain. It shows that the ground motion damage of slope has a cumulative effect. Since the shear strain of slope is not easy to measure, the rate of change of natural vibration frequency can be evaluated according to the acceleration response. According to the change of tangent slope, the development law of soil shear strain is judged and the stage of slope state is determined.

In the tests of group B and C, the slope body does not have a large displacement with a relatively low drop of frequency, and the rate of frequency drop is between 0 and 1%. The cumulative shear strain of slope is small and basically stable. With the increase in peak acceleration, the slope frequency decrease rate λ = 1 − 1.5%, and the cumulative shear strain of the slope is in a state of continuous and stable growth. When the frequency drop rate λ > 1.5 % or above, the slope displacement increases greatly, and a large area of sliding collapse begins to occur, and the slope body enters the failure stage III.

Although this test is not universal, the decreasing amplitude of natural vibration frequency will be different for different sliding body materials and different measuring point positions. Moreover, the model test has a size effect, and the measurement of natural vibration frequency of different measuring points will affect each other. However, as shown in Figure 23 and Figure 24, there is a strong positive correlation between the rate of frequency change and cumulative shear strain in the model test. The results show that it is appropriate to use the rate of change of natural vibration frequency as an index to measure the impact of vibration on slope. When it is applied to the actual slope, the distance between the measuring points is large enough and the mutual influence is small, and the change rate of natural vibration frequency of slope can reveal the change of macroscopic characteristics of slope more accurately.

## 4. Discussion

In this paper, the study on the dynamic response of high-steep slopes supported by anti-slide piles with different initial damage based on a shaking table test is completed. The rationality of taking shear strain as a slope deterioration development index is discussed, and the reliability of shear strain development near the measuring point can be judged by the rate of change of natural vibration frequency. It can provide early warning for the deterioration and development of each measuring point of slope under dynamic action in practical engineering and facilitate taking corresponding reinforcement measures. The comparison of each test parameter and the recommended threshold values are shown in the Table 6, Table 7, Table 8 and Table 9 below. The characteristic points of the acceleration amplification factor in Table 7 take the maximum value point J9 in the slope as the reference point, and the average value of all the measuring points in the sliding body is also processed to reflect the overall law of the acceleration amplification factor of the slope. In Table 8, due to the large difference of shear strain in the slope, considering that the local failure of the top and foot of the slope is far away from the pile, which has little effect on the overall stability of the slope, the maximum value of each copper belt of No.1, No.2, No.3 and No.4 is extracted, which can reflect the shear strain at different horizontal positions from the pile, and then the average value of the four maximum values can be used as a reference threshold for the overall shear strain of the slope. In Table 9, the decline rate of natural frequency is selected as the eigenvalue, and the maximum value and the average value of the sliding body are calculated as a comparison.

The deficiencies of this test and further improvement are as follows:

1. In order to ensure the similar dynamic properties of soil, this test only applies to the high-steep slope of gravel soil with a similar dynamic shear modulus ratio. For other types of slopes, laboratory model tests should also be carried out to verify the reliability.
2. Due to the small size of the test model, the distance between each position of the accelerometer is relatively close, and the measured results contain part of the overall damage information. Subsequent improvements should adopt larger model and further distance between measuring elements to reduce mutual interference between measuring elements.
3. In Section 3.5 of this paper, different working conditions of two measuring points are only given to illustrate the similarity between the change rate of natural vibration frequency and the development of cumulative shear strain, and to clarify the reliability of the change rate of natural vibration frequency as an indicator of slope deterioration. Subsequently, through laboratory tests or numerical simulation, the shear strain and natural vibration frequency changes at different positions under different initial damage degrees should be studied. The relationship between the development of shear strain and the rate of change of natural vibration frequency should be summarized.

## 5. Conclusions

Through comprehensive analysis of slope acceleration, shear strain and natural vibration frequency, the main conclusions are as follows:

1. Acceleration is not sensitive to the failure development process of deep soil below the slope surface. The shear strain energy of the slope measured by the phosphor bronze belt can reflect the failure process of the slope more accurately under dynamic action. Combined with the test phenomenon, the anti-slide piles of different qualities have an effect on the process of slope instability. When the quality of the anti-slide pile is intact, the failure development of the slope behind the pile is limited to the soil landslide at the top of the pile. The local slip of the shear belt in front of the pile mainly occurs at the foot of the slope. With the decrease in anti-slide pile mass, the failure of soil behind the pile changes into landslide, slope sliding and deep integral sliding. The failure in front of the pile develops into a local slip in the toe shear belt, and then the whole slip occurs along the slip belt.
2. By analyzing the white noise data of each acceleration sensor inside the slope, the frequency variation nephogram of the slope after loading at all levels can intuitively reflect the damage location of the slope. The soil behind and at the foot of the pile have the largest frequency variation.
3. There is a positive correlation between the rate of frequency change and cumulative shear strain, indicating that the rate of natural frequency change is an appropriate indicator to measure the impact of vibration on the slope. It can be used for the actual slope to reveal the variation law of macroscopic failure characteristics of the slope.
4. The threshold value of the natural frequency change rate can distinguish different development stages of the slope; 1% is the threshold value of stage II, and 1.5% is the threshold value of stage III.
5. In the follow-up research, the damage degree of piles and soil can be quantified by using parameters related to natural vibration frequency, and combined with numerical simulation software to be extended to more working conditions, to provide conditions for the prediction of the deterioration damage of slopes under earthquake action.

## Figures and Tables

**Table 1:** Similarity ratio of model’s physical parameters.

	Similarity Parameter	Similarity Reference Formula	Similarity Ratio
Main factor	physical dimension l	$C_{l}$	70
	gravity γ	$C_{\gamma }$	1
	acceleration of gravity g	$C_{g}$	1
Geotechnical material simulation	stress σ	$C_{\sigma }=C_{l}$	70
	Shear modulus ratio $G/G_{\max}$	$C_{G/G_{\max}}$	1
	shearing strain ϒ	$C_{ϒ}=1$	1
	damping ratio λ	$C_{\lambda }=1$	1
Micro-concrete structure	linear displacement S	$C_{S}=C_{l}$	70
	bending moment M	$C_{M}=C_{\sigma }C_{l}^{3}$	$70^{4}$
	shear force F	$C_{F}=C_{\sigma }C_{l}^{2}$	$70^{3}$
	strain ε	$C_{\varepsilon }=1$	1
Vibration wave loading	acceleration a	$C_{a}$	1
	time T	$C_{T}=C_{l}^{0.5}$	8.37
	frequency ω	$C_{\omega }=C_{l}^{-0.5}$	0.12

**Table 2:** Model pile stiffness reduction (relative to group A).

Test	Cracks Arrangement	Stiffness (Relative to Group A)
Group B	5 mm + 15 mm (local)	21.6%
	5 mm + 5 mm (local)	51.2%
Group C	8 mm hollow holes (overall)	99.9%
	5 mm + 15 mm (local)	21.5%
	5 mm + 5 mm (local)	51.1%

**Table 3:** Design parameters of anti-slide pile strength.

Cross-Section Parameter	Concrete Strength (MPa)				Reinforcement Parameter			Flexural Capacity
Cross-Section Area (mm^2^)	$f_{c}$	$f_{t}$	$f_{y}$	$f_{y}{}^{\prime }$	Tiepiece number	Ribbing number	Diameter d(mm)	design value (N·m)
50 × 36	16.7	1.57	300	300	3	2	2	119

Note: The above four parameters refer to the <<Code for design of concrete structures>> (GB 50010-2010). $f_{c}$ is the design value of the axial compressive strength of the concrete, $f_{t}$ is the design value of the axial tensile strength of the concrete axis, $f_{y}$ is the design value of the tensile strength of the reinforcing bar, and $f_{y}{}^{\prime }$ is the design value of the compressive strength of the reinforcing bar.

**Table 4:** Physico-mechanical indexes of model materials.

Medium		Gravity (kN/m^3^)	Cohesive Force (kPa)	Friction Angle (°)	Moisture Content (%)
Slipping body	model	22	8	42	9.8
Slipping belt	model	——	1.5	20	——
bedrock	model	22	2 × 10^7^	45	1

**Table 5:** Loading sequence.

Loading Sequence	Loading Waveform
1	White Gaussian Noise
2	0.1 g 5 Hz sine wave
3	White Gaussian Noise
4	0.2 g 5 Hz sine wave
┇	┇
Loading to failure	0.6 g 5 Hz Loading to failure

**Table 6:** Failure mode.

	Failure Mode
A	Tensile cracks on the top of the slope→Surface slip
B	Tensile cracks on the top of the slope→the failure of the slope behind the pile over the top
C	Tensile cracks on the top of the slope→Formation of deep slip surface→slope collapsed
Comparison	The smaller the damage of anti-slide pile, the more surface development of slope failure. With the increase in initial pile damage, the slope damage gradually moves down along the depth while the slope surface slips and collapses.
Reason	When the pile is not damaged, the pile can provide sufficient anti-sliding force, and the displacement of the pile is very small. The slope can only produce sliding surface along the slope surface from the top of the slope, and the overtop failure occurs. When the pile is damaged, when the seismic load is large, due to the decrease in the bearing capacity and the gradual increase in the displacement of the pile, the soil below the slope is loose, the shear stiffness decreases, and the shear strain and displacement increase gradually, forming deep sliding.

**Table 7:** PGA amplification coefficient.

	PGA Amplification Coefficient (PGA of J9 and Average PGA of Sliding Mass)
A	stage II peak value (0.6 g1) is 1.31, Average PGA of sliding mass is 1.19 stage III peak value (0.6 g3) is 1.41, Average PGA of sliding mass is 1.23
B	stage II peak value (0.5 g) is 1.16, Average PGA of sliding mass is 1.10 stage III peak value (0.6 g1) is 1.22, Average PGA of sliding mass is 1.16
C	stage II peak value (0.4 g) is 1.08, Average PGA of sliding mass is 1.05 stage III peak value (0.5 g) is 1.19, Average PGA of sliding mass is 1.12
Comparison	With the increase in magnitude, the acceleration amplification coefficient changes from a linear increase to a S-shaped change that first decreases, then increases and then stabilizes. With the increase in initial damage, the maximum acceleration amplification factor threshold of slope decreases gradually, and the average acceleration amplification factor threshold of landslide decreases gradually.
Reason	When the pile body is intact, it can provide enough anti-slide force, and the deformation of the pile body is small. Under the action of seismic waves, the natural vibration frequency of the slope is gradually close to the carrier frequency, resulting in frequency band coupling effect, resulting in PGA gradually increasing. When the pile body is damaged, when the slope is subjected to seismic load, compared with the intact slope, it is subjected to repeated shear, and the friction energy dissipation is larger. When the damping is relatively intact, the PGA is also larger. Therefore, when the damping is relatively intact, the PGA decreases.

**Table 8:** Shear strain.

	Shear Strain (1#, 2#, 3#, 4# Copper Strip, Take the Maximum Value of Shear Strain for Each Copper Strip, and Then Calculate the Average Value)
A	stage II threshold value (0.6 g1) is 49.817 με stage III threshold value (0.6 g3) is 112.301 με
B	stage II threshold value (0.5 g) is 43.516 με stage III threshold value (0.6 g1) is 97.975 με
C	stage II threshold value (0.4 g) is 57.865 με stage III threshold value (0.5 g) is 103.521 με
Comparison and Advice	When the quality of the anti-slide pile is intact, the failure development of the slope behind the pile is limited to the soil landslide at the top of the pile. The local slip of shear belt in front of pile mainly occurs at the foot of slope. With the decrease in anti-slide pile mass, the failure of soil behind pile changes into landslide, slope sliding and deep integral sliding. The failure in front of pile develops into local slip in the toe shear belt, and then the whole slip occurs along the slip belt. Suggested threshold for maximum average shear strain of stage II: 45 με Suggested threshold for maximum average shear strain of stage III: 100 με
Reason	When the pile is damaged, the stress deformation of the pile increases gradually, the displacement of the pile increases, and the displacement of the soil below the slope increases. When the slope is subjected to seismic load, the shear modulus of the soil below the slope behind the pile decreases faster than that when it is intact, so the shear strain gradually tends to the deep.

**Table 9:** Natural frequency.

	Natural Frequency (Maximum Frequency Decline Rate and Average Frequency Decline Rate of Sliding Mass)
A	
B	stage II maximum value (0.5 g) is 2.1%, Average Frequency decline rate of sliding mass is 1.12% stage III maximum value (0.6 g1) is 2.1%, Average Frequency decline rate of sliding mass is 1.47%
C	stage II maximum value (0.4 g) is 1.08%, Average Frequency decline rate of sliding mass is 0.97% stage III maximum value (0.5 g) is 1.62%, Average Frequency decline rate of sliding mass is 1.3%
Advice	Because there is a mutation in the frequency decline rate in the slope, but it cannot explain the overall stability, it is suggested that the average reference frequency decline rate is used as a threshold reference index. Suggested threshold for Average Frequency decline rate of stage II: 1% Suggested threshold for Average Frequency decline rate of stage III: 1.5%

## Data Availability

The data reported in this article are available from the corresponding author upon request.
